# Are Modern Head-Mounted Displays Sexist? A Systematic Review on Gender Differences in HMD-Mediated Virtual Reality

**DOI:** 10.3389/fpsyg.2020.01604

**Published:** 2020-08-07

**Authors:** Simone Grassini, Karin Laumann

**Affiliations:** Department of Psychology, Norwegian University of Science and Technology, Trondheim, Norway

**Keywords:** virtual reality, simulator sickness, cyber sickness, gender, sex

## Abstract

Modern head-mounted displays (HMDs) are a promising technology. Thanks to their affordable cost and versatility, HMDs are gaining attention from different sectors. However, the experience reported by the users of these technologies is sometimes negative. A number of people, when using an HMD, complain of various types of physical discomfort as well as symptoms like headache, disorientation, and nausea. These symptoms, developed during or after exposure to virtual environments, are commonly referred to with the term simulator sickness. Some scientific studies have shown that women are commonly more sensitive to simulator sickness. However, a gender imbalance in the susceptibility to simulator sickness has not been widely studied in the context of modern HMDs, and the studies that have been done have reported heterogeneous findings. The present systematic review aims to gather the pieces of evidence that support and oppose a gender difference in the susceptibility of simulator sickness in the framework of modern HMDs. We also aim to individuate other gender differences in the experience of the use of these technologies to establish whether there is sufficient evidence to support a gender discrepancy in the user experience.

## Introduction

Modern head-mounted displays (HDMs) are a promising technology. They are affordable and can induce a high sense of presence in the users (Shu et al., 2019). This phenomenon provides an enhanced level of immersion in virtual reality (VR) compared to traditional visualization means (e.g., LCDs). These tools are becoming more commonly used for entertainment as well as in research and for medical applications (Jerdan et al., 2018). However, the experience reported by the user is not always positive, and VR experience was found to influence human physiology (see Grassini and Laumann, 2020). A number of users, when exposed to HMD-mediated VR, complain of negative symptoms such as headache, disorientation, and nausea (Kennedy et al., 1993; Duzmanska et al., 2018). These symptoms are commonly referred to with the term simulator sickness (SS). In 2017, a scientific article proposed the bold idea that the effect of modern HMD-mediated VR may have “sexist effects,” referring to the higher incidence of SS in women compared to men (Munafo et al., 2017). In this context, with the term “sexist,” we aim to refer to a disadvantage on the use of the HMD technology that is specific for one gender over the other.

In the framework of the crucial inquiry of gender differences in the use of modern technologies, we aim to investigate gender discrepancies in SS symptoms and possible gender differences in other factors of the VR experience that may be “sexist” (e.g., learning ability and performance in VR). The present study aims to answer the following research questions: (1) What evidence exists in the current scientific literature for a gender difference in experiencing modern HMD-mediated VR? (2) Is there sufficient evidence for a gender imbalance to define such technology as “sexist”?

### SS and Gender Differences

Notwithstanding the effort undertaken by the headset producers and the software developers to minimize the adverse effects from the use of the technology, SS still poses a problem to the widespread adoption of VR (Lang, 2016). Only a handful of experiments have explicitly investigated the negative symptoms that arise from the use of modern HMD-mediated VR (e.g., Munafo et al., 2017). HMDs are not a new concept, and the modern HTC Vive and Oculus products are only the latest, improved version of more than 50 years of ideas (Sutherland, 1968; Mazuryk and Gervautz, 1996; Choi et al., 2015). However, previous generations of HMD were not widely available in the market, and users were reluctant to accept those devices due to the high price and limited applications. On the contrary, modern HMD devices have received widespread attention, first from the gaming industry and lately from a wider audience of multimedia users.

It is worth noting that SS is not the only side effect of modern HMD technologies. Some VR users report an increase in hardware-related symptoms, such as headache and eyestrain, while expressly reporting no experience of SS (e.g., Stanney and Salvendy, 1998; Munafo et al., 2017; Curry et al., 2020). The methods of administering the questionnaires for assessing SS are also sometimes sub-optimal: while the questionnaires are commonly given right after a VR exposure, the negative symptoms related to SS can, in fact, be developed not only immediately after the VR experience (Merhi et al., 2007; Stoffregen et al., 2010) but also up to 12 h after the exposure to the environment (Stoffregen, 1985).

SS has been often associated with motion sickness (MS), that is, the feeling of discomfort that many people experience, for example, while using a bus or a ferry (Kennedy et al., 1992). Several scholars have argued that SS may be a type of motion sickness while other scholars argue that these are connected but separate conditions (see e.g., Mazloumi Gavgani et al., 2018). The term “simulator sickness” has been historically created to define the effects induced by simulators and has been later adapted to non-simulator virtual experiences. Sometimes in the literature, the terminology “Cyber Sickness” (CS) is also used. Some scholars have attempted to differentiate CS from SS (see e.g., Stanney et al., 1997; Kennedy et al., 2003). Recent developments have suggested that the use of the term CS may be appropriate referring to the symptoms experienced when the person's visual input is completely computer-generated, as in the HMD-mediated VR (see e.g., Duzmanska et al., 2018); however, there is now no consensus on which terminology should be used in relationship to modern VR technologies. Generally, both SS and CS are being used in the scientific literature to describe the unpleasant ill symptoms evoked by the use of various types of VR technology (e.g., Bruck and Watters, 2011; Serge and Moss, 2015; Lee et al., 2017). In the present article, for simplicity, we decided to use the terminology “Simulator Sickness” referring to SS and CS.

Anecdotal reports as well as controlled experimental investigations have reported a gender difference regarding the degree of SS symptomatology, with females being more sensitive to SS (Munafo et al., 2017). As SS is considered to be a phenomenon closely linked to MS, results from studies investigating MS have been generalized to SS. A predisposition for MS for women has been consistently shown in several studies. For example, when investigating seasickness, women were found more susceptible than men in a ratio of around 5 to 3 (Lawther and Griffin, 1988). Several studies have evaluated the possibility of empirically proving the generalization of the female susceptibility to MS to visually induced SS in controlled laboratory experiments; however, the results have been mixed (see Munafo et al., 2017).

The motivations behind sex imbalance on SS symptoms remain unknown. Nevertheless, some scientists have tried to explain those differences with hormonal levels during the female menstrual cycle. Clemes and Howarth (2005) showed that females are more sensitive to SS, especially on day 12 of their cycle (please note that women were also more susceptible than men on days 5, 19, and 26, and not just on day 12, of their menstrual cycle). The authors proposed that this effect may be linked to estradiol. However, the authors reported that the increased SS susceptibility due to menstrual cycle was short lived (24–48 h). Such a phenomenon may be responsible for increasing the average SS scores for the female participant group. Many researchers have criticized this idea (see Biocca, 1992; Pausch et al., 1992). Biocca (1992) alternatively proposed that SS susceptibility may have to do with the fact that females generally have a larger field of view (FOV), and a larger FOV is associated with an increased susceptibility to SS. Furthermore, SS is generally evaluated based on self-reports, and thus, it has also been proposed that males may be more likely to underreport the level of subjective discomfort compared to females (e.g., Biocca, 1992; Kolasinski, 1995). Other studies have suggested that the gender imbalance in SS may be related to sex differences in cognitive functions (e.g., Voyer et al., 1995; Kimura, 1997; Giammarco et al., 2015) or sexual dimorphism (the fact that males and females are physically different; see Munafo et al., 2017) and its consequences on the stability of movements (in connection with the postural instability theory of motion sickness, which suggests that the body instability in an new, unusual situation like in VR, promote SS; see Riccio and Stoffregen, 1991).

## Methods

We conducted a systematic literature review search to identify publications in journals and conference proceedings. The themes of interest of the search were gender differences in the experience from the use of modern HMD-mediated VR. We conducted the search according to the Preferred Reporting Items for Systematic Reviews and Meta-Analysis (PRISMA) guidelines (Liberati et al., 2009). The research terms used for the literature search were the following: “male” AND “HMD,” “female” AND “HMD,” “gender” AND “HMD.”

We only considered literature in English for the present review. We performed the literature search on February 14, 2020, and included articles published from 2016 to present. We chose 2016 because it was the release year of the Oculus Rift CV1, one of the very first commercially released, consumer-oriented modern HMDs. The HTC Vive was also commercially released in 2016. Furthermore, 2016 has been defined as “a golden year for virtual reality” (Gaggioli, 2017) due to the growth in popularity that the technology reached in that year due to its commercialization to retail customers. However, it is worth noting that both of the major development companies (Oculus and HTC) had released previous development kit versions of their HMD (Oculus since mid-2013, and HTC in August 2015). However, the development kit versions of the HMDs have also been widely used in the analyzed literature body from 2016 and onward.

We searched Scopus, Web of Science, and IEEExplore. We chose the first two databases for their multidisciplinary focus, which fits the topic of the present investigation. We selected IEEExplore for the focus on technology developments of the IEEE conferences. The keywords were searched using title+abstract+keywords option on Scopus, topic option (title+abstract+keywords) on Web of Science, and all metadata in IEEExplore.

No other limiters were inserted in the database searches. We imported the entries (294 articles) into EndNote and removed duplicates. A few more articles (6) were added from previously known articles on the topic and from the relevant references from those. One of these articles was added under suggestion during the peer-review phase of the present manuscript. After duplicates were removed, there were 198 articles. We first screened titles and keywords from the remaining articles, and we eliminated those that we considered outside the topic of interest (e.g., did not have anything to do with HMDs or VR). The articles that remained after this phase were 95.

**Figure 1:**
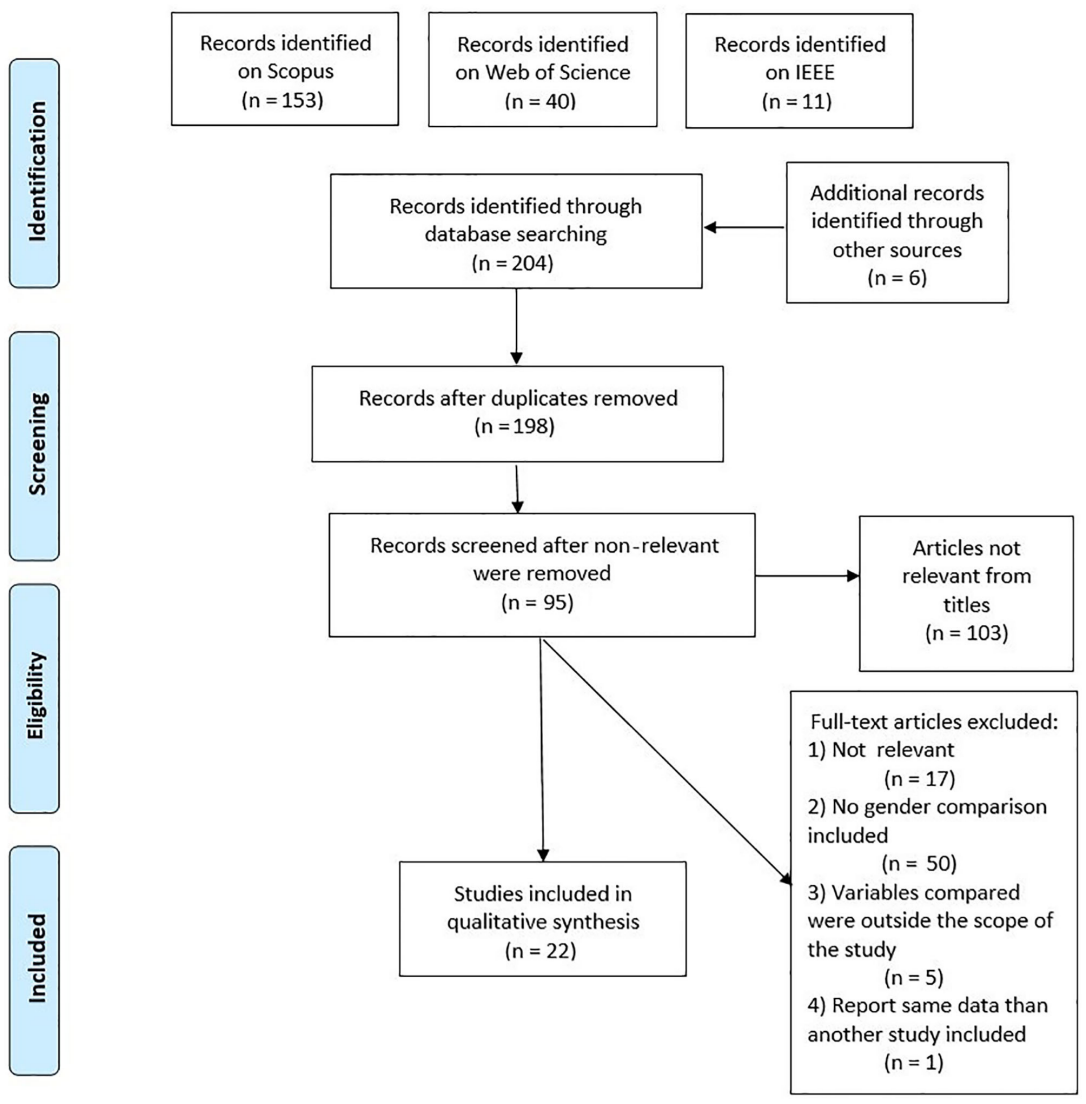
Overview of the article selection process.

We included these articles that we found to fit the topic of interest for full-text evaluation. In this phase, we excluded the following types of articles from further analysis: articles that did not report first-hand experimental data and articles that were not relevant for the topic of interest (e.g., from a completely different field, from full-text evaluation, *n* = 17); articles that fit the topic but did not include an explicit (and statistically verified) comparison between genders (*n* = 50); and articles that analyzed gender difference variables outside the interest of the present study (e.g., gender tastes, *n* = 5). One article was excluded as it reported the same data of an article already included in qualitative synthesis. We included articles that reported more than one experimental finding, using different samples of participants, for each sample separately in the final analysis table. In Table 1, we also included articles that utilized more than one visualization method (e.g., 2D screens, etc.) in addition to VR, even if they did not report gender differences separately for each condition. A PRISMA flow-chart is presented in Figure 1 to summarize the various steps of article selection for the present review. We included a total of 22 papers in the present systematic review.

**Table 1:** List of studies included from the literature review.

**References**	**Study *N* (M/F)**	**Mean age (*SD*)**	**Head-mounted display**	**Virtual environment**	**Locomotion**	**Induced level of SS, questionnaire or method of assessment used**	**Main gender-related results**	**Virtual environment details**
Allen et al. (2016)	73 (28/45)	20.47 (6.07)	Oculus DK1	MT; LE	Stationary	ne, MSAQ (Gianaros et al., 2001). Evaluation of “quitters” vs. “survivors”.	F were more likely than M to feel discomfort and terminate the simulation; F performed better than M in the velocity stereovision task; no difference between F and M in the other performance measures	Four tasks designed to measure static and dynamic stereovision performances
Robert et al. (2016)	14 (9/5)	26.1 (3.1)	Oculus DK2	RE; LE	Stationary	none	No significant difference between genders on all postural tests	Recreation of a laboratory environment
Cárdenas-Delgado et al. (2017)	89 (43/46)	Divided into two subsamples: 26.38 (3.87) and 25.38 (4.11)	Oculus DK2	RE; LE	Physical bicycle	none	No memory performance difference in VR between genders	Virtual maze navigation developed to assess spatial short-term memory
Munafo et al. (2017) – Experiment 1	36 (18/18)	20.72 (0.85)	Oculus DK2	Game; LE	Stationary	Design with multiple estimation of SS, SSQ—mean non reported (around 40, estimated from the reported Figure 1), high level of induced SS.	M and F did not differ with regard to the degree of simulation sickness	Balancer Rift (share.oculus.com); VR-version of the classic balance board game in which players need to guide a ball toward an exit
Munafo et al. (2017) – Experiment 2	36 (18/18)	22.72 (3.56)	Oculus DK2	Game; HE	Controller	Design with multiple estimation of SS, SSQ—mean non reported (around 70, estimated from the reported Figure 1), high level of induced SS.	F experienced a higher level of SS compared to M. Sex differences in susceptibility to motion sickness were anticipated by gender differences in body sway.	Affected (games.softpedia.com); horror-game including jump-scares
Wilson and Kinsela (2017)	29 (15/14)	19.96 (nr)	ProView TM XL 50 HMD	MT; LE	Stationary	ne, MSSQ-S (Golding, 2006).	No differences between M and F in SS; however, F reported a higher level of SS before the experiment compared to M	Modification of an object location task to challenge the participant's visual-vestibular interaction.
An et al. (2018)	21 (12/9)	20.58 (1.06)	Oculus CV1	RE; HE	Stationary	None	HMD VR has a positive effect on learning outcomes for M but not F	Military training scenarios
Juan et al. (2018)	40 (20/20)	26.52 (7.57)	(a) Google Cardboard V2; (b) Samsung Gear VR Innovator Edition (SM-R320)	RE; LE	Stationary	None	Gender does not affect the use of low- vs. high-quality HMD	Visualization of food on a dish
Melo et al. (2018)	128 (64/64)	21.52 (3.878)	Oculus DK2	RE (both real and computer-generated); LE	Stationary	SSQ. Mean values are not presented, and it is not possible to estimate them.	No gender difference in cybersickness nor sense of presence; F experienced a higher sense of reality for the computer-generated scene	Captured (camera) and synthesized (computer-generated) 360-degree images
Mousas et al. (2018)	72 (56/16)	23.24 (5.18)	Oculus DK2	RE; HE	Controller	None	F showed a higher level of emotional sensitivity compared to M	Encounter with simulated characters (realistic or zombie-like)
Roettl and Terlutter (2018)—Experiment 1	234 (100/134); 73 in the VR condition (no M/F ratio specified)	24.52 (4.13)	Oculus Rift headset (no model specified), also 2D, and 3D (large 46-inch, stereoscopic 3D-capable television)	RE; HE	Controller	None	No difference between M and F in information recognition and recall	A “jump'n'run” video game, designed and developed in 2D and 3D, as well as for HMD; the authors claimed that spatial depth perception is possibly relevant for game play
Roettl and Terlutter (2018)—Experiment 2	53 (16/37); 15 in the VR condition (no M/F ratio specified)	nr	Same as Roettl and Terlutter—Experiment 1	RE; HE	Controller	None	No difference between M and F in cognitive load (measured with a memorization-retrieval task pre and post experiment)	Same as Roettl and Terlutter—Experiment 1
Khashe et al. (2018)	100 (34/66)	nr	Oculus DK2, and flat-screen PC	RE; LE	Controller	None	No gender difference for the sense of presence, behavior, or performance in VR	Pro-environmental request in a simulated photorealistic office
Scheibler and Rodrigues (2018)	46 (27/19)	nr	nr	Game; HE	Controller	SS was evaluated using a 17-item form.	F reported to experience sense of embodiment in both first- and third-person game; F presented discomfort in first-person perspective, but not in the third-person one	Cartoonish running/obstacle game in first and third person
Rangelova and Marsden (2018)	72 (54/18)	25.28 (5.16)	Oculus DK2	RE; HE	Controller	Evaluation of objective measure (time spent in the simulation)	F spent less time in VR; F did not want to repeat the experience as much as M. However, no significant gender differences in enjoyment and regarding how interesting they considered the driving experience in the simulation.	VR driving simulator
Al Zayer et al. (2019)	28 (14/14); note that two F quit the experiment from the original sample of 30, due to severe discomfort	23.04 (3.59)	HTC Vive	RE; LE	Controller	SSQ, 51.29 in the normal, no-restriction mode, and 35.53 in the FOV restriction mode. High level of induced SS in both modes.	No significant differences between M and F in SS; no gender difference for sense of discomfort scores	Natural scenery, adapted version of the Rocky Hills Environment—Light Pack asset from the Unity Asset Store
Bracq et al. (2019)	29 (8/21)	nr	HTC Vive	RE; LE	Stationary	SSQ, two different experimental groups, from 2.97 to 3.94 average score. Low level of induced SS.	No gender differences for workload, immersion, or SS	Medical-oriented training scenario: training and instrumentation surgery table
Chang et al. (2019)	100 (39/61)	20.28 (2.05)	HTC Vive	RE; HE	Stationary	None	F were more willing to use the HMD version of the simulator (instead of the screen one); M experienced a higher sense of presence using HMD	Gynecology training (IFOREAL)
Clifton and Palmisano (2019)	25 (13/12)	23.92 (5.25)	HTC Vive	RE; LE	Physical walk and controller (no difference in SS among the two conditions)	SSQ. Does not report total SSQ scores.	No gender difference for SS; the incidence of SS was relatively high in both sexes	A 2-min introduction to the natural environment walk simulator “Nature Treks VR”
Liang et al. (2019)	25 (15/19)	21 (1.2)	Oculus CV1	RE; LE	Controller	None	F were better in localization and memory tasks	Navigating in a three-floor building
Moroz et al. (2019)	19 (10/9)	26.5 (nr)	Oculus CV1	MT; LE	Stationary	SSQ, descriptive data was not reported.	F reported more SS	Conflict detection task, and visual speed-discrimination task
Narciso et al. (2019)	128 (63/65)	21.02 (4.604)	Oculus DK2	RE (2D and 3D); LE	Stationary	SSQ. Data are reported separately for male and female, while aggregate data are not reported. For the 2D video, male SSQ averaged 8.46, and female 12.47. For the 3D video male SSQ averaged 10.72, while female averaged 8.01. Overall the induced SSQ of the simulation they used was low.	F scored higher spatial presence, realness, and overall presence in the 3D condition while M achieved a higher spatial presence, realness, and overall presence in the 2D condition; F participants reported higher nausea in the 2D condition, while M reported higher nausea in the 3D condition	Video, exploration of urban scenes
Shafer et al. (2019)	160 (94/66)	20.5 (nr)	Oculus DK2 and CV1	Game; HE	Controller	SSQ. 6.62 average for men, and 10.42 average for women and. Low level of induced SS.	F experienced significantly higher levels of SS than M across conditions	Three different games: Minecraft, Elite: Dangerous, and Lucky's Tale
Curry et al. (2020)	79 (38/41)	21.84 (4.19)	Oculus CV1	Game; HE	Controller for drivers (or watch only for passengers)	SSQ. 38.58 for men, and 40.41 for women. High level of induced SS.	No SS differences between M and F. (however, among drivers, F discontinued the simulation earlier)	Car driving game (Assetto Corsa by Kunos simulations). Participants either have an experience as drivers or as passengers.

*The columns (from left to right) are study authors, year of publication, N (male/female), mean age (SD), head-mounted device (HMD) used, evaluation of the type of displayed environment, principal results regarding gender reported in the study, and details on the utilized environment*.

*The following abbreviations were used: MT, minimal/cognitive task; RE, realistic environment; LE, low emotion/arousal; HE, high emotion/arousal; 2D, two-dimensional; 3D, three-dimensional; M, males; F, females; VR, virtual reality; nr, nonreported (or impossible to evaluate); SS, simulator sickness; ne, not evaluated*.

### Table Categorizations of Virtual Environments

We categorized three types of content in the studies included for analysis: minimal/cognitive task (“MT” in Table 1), game, and realistic (including 360-degree videos). Each category refers to a different set of features in the virtual environment. Minimal/cognitive task environments were those that presented basic shapes, textures, and colors (commonly used in cognitive tasks), with very simple interactions with the users and a low level of photographic realism. The “game” category included those environments where the user could actively interact and perform tasks in the VR. This category included software developed for experimental purposes, as well as commercially available videogames. In the realistic category, these environments were created to have reality-like features, independent of the task the user had to perform. Furthermore, we categorized each environment with regard to the extent that it might have stimulated emotions or arousal in the user. This category also included the environments created using 360-degree cameras or environment displaying images or video with the possibility for the user to have a 360-degree view. High emotion (HE) environments possibly promoted emotional reactions (e.g., warfare games, VR including fearful stimuli, etc.), while low emotion (LE) environments did not contain elements that may have had a major effect on the users' emotions (e.g., cognitive tasks, walking scenario, etc.). The categorization of virtual environments used was inspired by the one used in by Saredakis et al. (2020).

The locomotion type experienced in the simulation has often been studied in relation to SS. The SS-inducing properties of virtual locomotion are commonly accepted in the VR community and often reported in the literature (Bruder et al., 2012; Nilsson et al., 2018). A different level of experienced SS in locomotion vs. non-locomotion environments is supported by evidences from the study of MS (e.g., people at sea get more MS when the sea is more turbulent). We have included into the category “stationary” all those environments that did not allow “non self-locomotion” (e.g., where the participants could explore the environment only moving its head and where the environments did not produce movements alone). All the other environments were categorized based on the way locomotion could have been achieved by the participant (usually via a controller, as, e.g., in the case of car-driving simulators).

In the following table, we have also specified which questionnaire was used to evaluate SS, and if the overall level of SS provoked by the scenario was high or low, as we hypothesized that the level of provoked SS may be a sensible variable to examine in relationship with gender. To establish if the environment was highly or lowly provoking SS, we interpreted the values of the SSQ (the most widely used questionnaire in the reviewed studies) in light with the findings of the comprehensive meta-analysis on SS in HMD-mediated VR of Saredakis et al. (2020). In their meta-analysis, Saredakis et al. (2020) reported 28 for the total SSQ, as pooled mean among all the studies they analyzed. We used this reported mean as reference to divide the study analyzed in the present article as low (<28) or high (>28). Please note that previous articles have tried to interpret the degree of experienced SS differently. According to Kennedy et al. (2003), SSQ total scores between 10 and 15 indicate significant symptoms, while those between 15 and 20 are concerning and over 20 indicate a problem simulator (see Saredakis et al., 2020). However, different from laboratory studies using HMDs, as the one reviewed by Saredakis et al. (2020), these estimations were taken from military personnel using flight simulators. Due to the technology used in the studies of the present review, and the analogy of study participants, we decided to rely on the average score reported by the review of Saredakis et al. (2020). We decided to categorize only those studies using the SSQ as evaluation for SS, and that reported the total SSQ value. In case the study uses a pretest posttest design for evaluating the final SSQ (as e.g., according to the recommendation of Regan and Price, 1994; Wilson and Kinsela, 2017), the posttest (after exposure) reported SSQ values were used. Studies that used a non-standard version of SSQ (e.g., removing items) were excluded from the categorization.

See the Table 1 note for a summary of the selected contents and an overview of the abbreviations.

## Results

This review contained 24 experiments from 22 different scientific articles. An overview of these studies, their methodologies, and their findings are reported in Table 1. Two of the included studies were published in 2016 (Allen et al., 2016; Robert et al., 2016), three in 2017 (Cárdenas-Delgado et al., 2017; Munafo et al., 2017; Wilson and Kinsela, 2017), eight in 2018 (An et al., 2018; Juan et al., 2018; Khashe et al., 2018; Melo et al., 2018; Mousas et al., 2018; Rangelova and Marsden, 2018; Roettl and Terlutter, 2018; Scheibler and Rodrigues, 2018), eight in 2019 (Al Zayer et al., 2019; Bracq et al., 2019; Chang et al., 2019; Clifton and Palmisano, 2019; Liang et al., 2019; Moroz et al., 2019; Narciso et al., 2019; Shafer et al., 2019), and one in 2020 (Curry et al., 2020).

The experiments were very heterogeneous regarding the number of participants tested, ranging from a minimum of 14 (Robert et al., 2016) to a maximum of 234 (Roettl and Terlutter, 2018). With the exception of a few studies (Munafo et al., 2017; Juan et al., 2018; Melo et al., 2018; Al Zayer et al., 2019), the other studies did not report a balance between male and female participants. Such imbalance may undermine the validity of these studies regarding the reported gender differences. However, due to the limited empirical studies on the argument, we decided to include all the studies independently on the participants' gender imbalance.

The population that participated in the analyzed studies was mainly composed of undergraduate university students, as some of the articles explicitly reported. The mean age was <30 years in every study; it was low as 19.96 years in the study of Wilson and Kinsela (2017).

Oculus and HTC products were used in the majority of the analyzed studies. Most of the studies until 2019 used the DK version of the Oculus Rift, while from 2019, the use of the market-ready releases of Oculus (CV1) and HTC Vive has become the standard. The smartphone-powered Samsung gear VR and Google Cardboard were used in one of the analyzed studies (Juan et al., 2018). One study employed the lesser known HMD ProView TM XL 50.

The virtual environments employed in the studies were remarkably diverse. Some were specifically designed to elicit a high level of emotion and arousal in the users (e.g., a horror-themed game and a warfare simulation), while others presented neutral content (e.g., cognitive tasks). With the categorization in Table 1, we attempted to simplify the understanding of the content of the VR environments and to understand how different types of environments may shape human experience.

**Figure 2:**
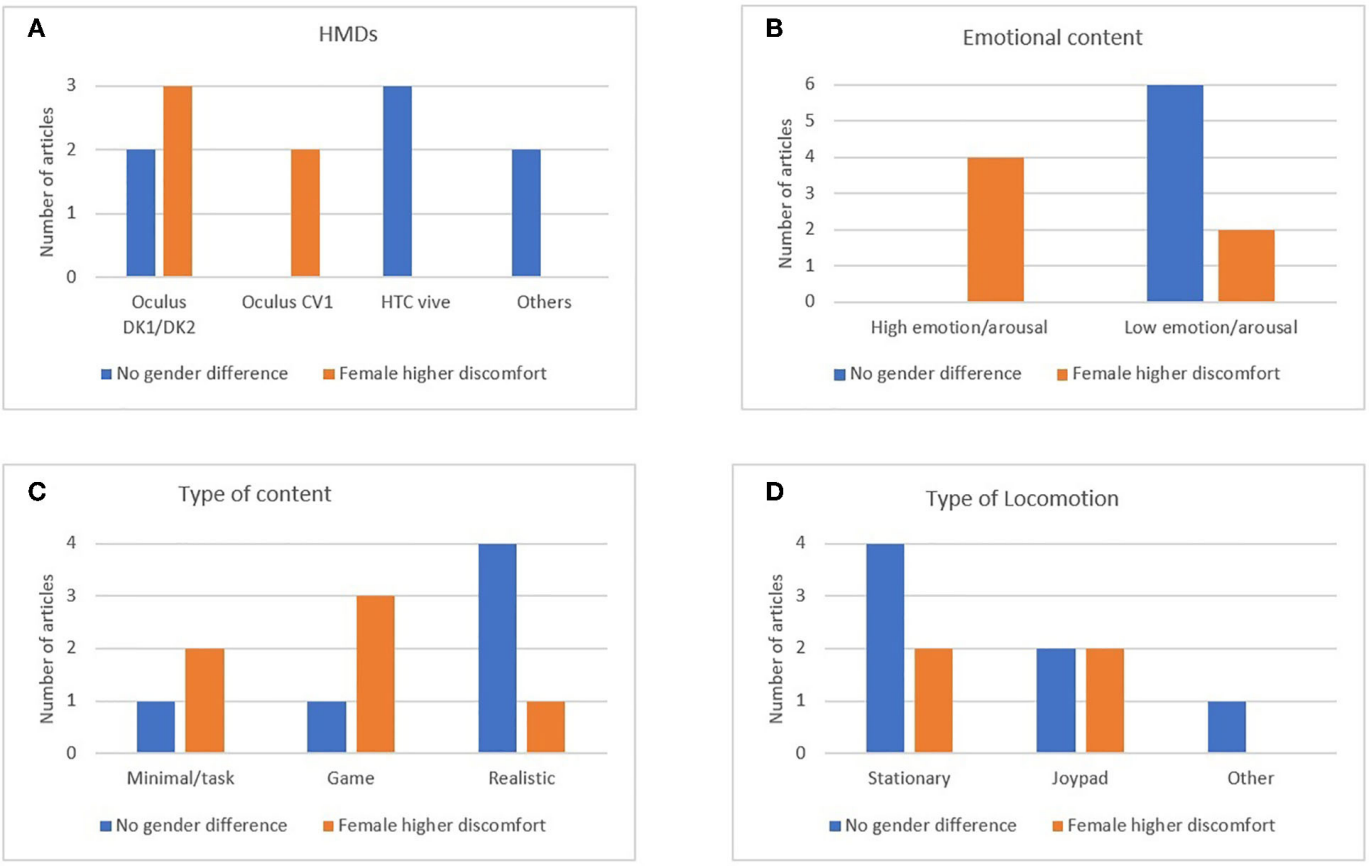
The graphs present the number of studies that reported a different level of discomfort between male and female participants, divided by **(A)** the type of head-mounted device (HMD), **(B)** the emotional level in the simulated environment, **(C)** the type of virtual reality content, and **(D)** the type of locomotion experienced in the virtual environment.

Of the 24 experiments included in the present review, 5 (Allen et al., 2016; experiment 2 in Munafo et al., 2017; Rangelova and Marsden, 2018; Moroz et al., 2019; Shafer et al., 2019) reported a higher susceptibility to discomfort in VR for female compared to male participants. Scheibler and Rodrigues (2018) reported that female participants experienced discomfort only in specific conditions (first-person perspective movements). Narciso et al. (2019) reported that males and females may be sensitive to different features of the simulated environment and experience discomfort (nausea) accordingly (females more for two-dimensional [2D] environments, while males for three-dimensional [3D] environments). Seven of the analyzed studies did not report differences in SS and discomfort during VR simulation between male and female participants (experiment 1 in Munafo et al., 2017; Wilson and Kinsela, 2017; Melo et al., 2018; Al Zayer et al., 2019; Bracq et al., 2019; Clifton and Palmisano, 2019). Figure 2 displays the studies that reported increased discomfort between males and females, divided by the type of utilized HMD equipment and categories of VR content. Only studies that reported a definite disadvantage for females regarding SS or discomfort in VR are included in the graphs [of the studies cited earlier, Narciso et al. (2019) was not included due to its ambiguous results regarding gender and VR discomfort].

Based on Figure 2, only studies employing Oculus HMDs have reported gender differences in the level of SS or discomfort. In studies with high emotional content—or a highly arousing environment—female participants experienced more discomfort compared to males. For most of the studies that employed minimalistic VR scenarios or cognitive tasks, as well as game environments, females were more susceptible to discomfort compared to males.

The use of stationary view (e.g., only allowing the exploration of a static environment with the movement of the head) or the use of a joypad to explore the virtual environment is reported as the most common way to interact with the VR. Notable exceptions were the study of Cárdenas-Delgado et al. (2017) that allowed the subject to move with the use of a physical bicycle, and Clifton and Palmisano (2019) that in one of their experimental conditions allowed natural walking to interact with the VR scene. Figure 2 (Table D) shows a summary of the number of studies for each locomotion methods reporting either gender balance or unbalance for SS.

We attempted to divide the study based on the induced level of SS, in high- and low-level SS. Almost all the studies that were possible to evaluate regarding such distinction showed to provoke a generally high level of SS in the participants. Studies reporting a high level of induced SS were (Munafo et al., 2017) (both reported studies), Al Zayer et al. (2019), and Curry et al. (2020). Of those, only Munafo et al., 2017 (study 2), reported a gender difference on the level of SS. The studies reporting a general low level of induced SS were Bracq et al. (2019) and Shafer et al. (2019), with the latter study reporting a gender unbalance on the level of SS.

With regard to other abilities measured in the reviewed articles, there were no gender differences in postural tasks during the simulation (Robert et al., 2016), in memory capacities employed during the simulation (Cárdenas-Delgado et al., 2017), in the experience of low- vs. high-quality simulation (Juan et al., 2018), in cognitive abilities as information recognition and retrieval as well as cognitive load during the simulation (Roettl and Terlutter, 2018), and in sense of presence and performance (Khashe et al., 2018). Rangelova and Marsden (2018) also found no significant gender differences in enjoyment and interest of the simulation.

Some studies reported that female participants experienced better performance or enhanced cognitive abilities in VR compared to males. Allen et al. (2016) found that female participants performed better in their VR cognitive task (velocity stereovision task); however, there were no performance differences in the other tested tasks. Furthermore, females performed better than males in localization and memory tasks (Liang et al., 2019).

One study found that female participants were more sensitive to emotional information mediated by fictional characters displayed in VR (Mousas et al., 2018). This phenomenon may be linked to a higher perception of realism or sense of presence experienced in the environment. Scheibler and Rodrigues (2018) also argued for a higher sense of presence in the simulated environment for female participants (sense of embodiment in a simulated character).

Female participants achieved a higher level of spatial presence, realness, and overall presence when HMD was used to show 3D images (Narciso et al., 2019). According to one study, females were also more willing to adopt the use of HMD VR compared to males (Chang et al., 2019).

One study (An et al., 2018) showed a performance advantage for males in cognitive tasks. Finally, the study of Narciso et al. (2019) found that males achieved a higher spatial presence, realness, and overall presence when HMD displayed images in 2D.

## Discussion

The present study aimed to investigate, using the currently published literature on the topic, gender differences with regard to experiencing HMD-mediated VR. Furthermore, the study aimed to understand whether there is sufficient evidence in the literature to define HMD technology as “sexist” due to its different effects on gender, as claimed by a previous study (see Munafo et al., 2017).

In this regard, we believe that it is important to clarify that Munafo et al. (2017), in their work titled “The virtual reality head-mounted display Oculus Rift induces motion sickness and is sexist in its effects,” claimed that the Oculus Rift, as a technology, is sexist in its effects. However, they specified that this does not mean that this was the intention of its designer; their call for the “sexism” of the technology is based solely on the disparate impact on women and men (Boyd, 2014).

The results presented in the present review showed that the majority of studies employing HMDs and explicitly reporting gender differences did not show any difference between male and female participants. It is possible that specific VR environments may promote female discomfort (e.g., games and environments where cognitive tasks were performed); however, this remains an open question due to the little published literature on the topic. Other possible sex differences between males and females (e.g., cognition and performance) did not differ between genders.

### SS: Males vs. Females

Contrary to some reports in the literature, and contrary to the findings especially reported for MS (Jokerst et al., 1999), the majority of analyzed studies did not report a higher incidence for SS in females compared to males. Seven studies explicitly reported no sex differences in SS, while only five reported that females experienced a higher degree of SS symptomatology. However, as already proposed by Munafo et al. (2017), some of the studies on VR-related discomfort did not directly assess SS. For example, Allen et al. (2016) did not directly evaluate the incidence or the severity of SS; they simply reported the number of “quitters” and “survivors” of an experiment. This measure does not take into consideration that people may want to terminate the experiment for reasons that are not related to SS (Merhi et al., 2007; Stoffregen et al., 2008; Koslucher et al., 2015). This fact is also true for other studies included in this review such as Rangelova and Marsden (2018), who only reported that female participants wanted to spend less time in the HMD simulation and did not want to repeat the simulation experience as much as males. Further, Czerwinski et al. (2002) only evaluated a general sense of “discomfort” without referring qualitatively or quantitatively to SS.

#### HMDs and Gender Differences

Figure 2 provides some indications on the role of HMDs or the content of the simulation on the difference between genders in SS. Figure 2A indicates that there have been reports of gender differences for Oculus HMDs (both the development kit and CV1). Previous studies have proposed that, since females can have a smaller interpupillary distance (the distance between the eyes, where the HMD lenses are positioned, hereinafter IPD; Fulvio et al., 2018), some types of HMDs may not be adjusted. Consequently, there would be side effects, such as eye strain and discomfort (Saredakis et al., 2020). Another technical factor (unrelated to gender) that affects the degree of SS is the FOV (e.g., Seay et al., 2001; Fernandes and Feiner, 2016). For the Oculus DK 2, the reported IPD range is between 52 and 78 mm (Yao et al., 2014), with a FOV of around 100 degrees. The Oculus CV1 is reported to have an IPD range between 58 and 72 mm, paired with a FOV of around 100 degrees horizontal. On the other hand, the HTC Vive has a smaller range, from 61 to 74 mm, and roughly the same FOV (for more technical info and comparison between the types of equipment, see the excellent overview reported by ArviVR, 2018). Taken together, these findings do not suggest a link between a female predisposition to develop SS or discomfort and the possibility of adjusting the IPD or FOV characteristics in particular types of equipment. Other differences between how these types of equipment display the images or interact with the software may be the culprit for the dissimilarity in SS susceptibility between males and females. However, this idea is purely speculative, and there is no evidence—at least currently—for it. It is also worth mentioning that most of the studies that have used Oculus equipment used development kits (DK1 and DK2), and not the final consumer-oriented version.

However, too few studies have utilized the Oculus CV1 or a later Oculus version; hence, it is not possible to draw a conclusion. The difference reported in Figure 2A regarding SS susceptibility and gender for different HMDs may be due to pure chance: the study sample is limited, and many other variables may be at play at the same time (e.g., study design and VR content).

Nevertheless, the problem of an insufficient IPD range in modern HMD technologies is worth considering. Based on the data of ANSUR II, large public datasets that report IPD measurements (see Paquette, 2009), the remarkable article of Heaney (2019) showed that the modern Oculus Quest's mechanical IPD adjustment makes it most suitable for around 99% of men and 93% of women. Furthermore, the HMD Rift S, which employs non-adjustable fixed lenses, could optimally suit only 46% of men and 43% of women. Heaney (2019) indicated a small (but still notable) difference in suitability of the Oculus HMDs between males and females. However, please note that Heaney (2019) article was published on the VR-related news website “uploadvr.com” and not in a peer-reviewed scientific journal.

Saredakis et al. (2020) performed a comprehensive systematic review and quantitative meta-analysis focused on factors that correlate with SS. They attempted to quantitatively estimate the female susceptibility to SS, correlating the percentage of females in studies and total Simulation Sickness Questionnaire (SSQ) scores reported in the analyzed studies. However, the authors explained that it was not possible for them to perform a more accurate quantitative analysis because the vast majority of the published studies have not supplied data breakdowns by sex. Their review found no significant associations (nor a tendency, *r* = −0.172, *p* = 0.170, as reported in their study) between SSQ total score and gender.

Female participants may also be more willing to report distress and pain (Barsky et al., 2001; Meulders et al., 2012). This may be due both to social biological differences and social expectations (Barsky et al., 2001).

#### Emotional/Arousing Environment and Gender Differences

Articles that displayed environments that induce high emotions/arousal in the participants more often reported female users who experienced a higher level of discomfort vs. males (Figure 2B). These results suggest that a higher level of arousal, emotion, and/or stress may increase the female susceptibility to SS and discomfort, in general, during VR.

General differences in personality between men and women may explain why females experience a higher sense of discomfort in emotional/arousing simulations. Women (especially young women) have a higher level of neuroticism compared to men (for a meta-analysis, see Jorm, 1987). This gender-related personality difference may be one of the reasons for the higher level of SS experienced by women. The current literature on the topic has shown a relationship between neuroticism and human health (e.g., Charles et al., 2008; Lahey, 2009). Researchers have proposed that neuroticism modulates psychological and physiological response to stress. The interaction between the VR environment and personality traits in determining the response to stressors (and therefore SS and discomfort symptoms) may be the reason why most of the studies where emotional and arousing stimuli are presented also report that women have a higher sense of discomfort or SS. Converging evidence from behavioral and psychophysiological studies support the idea that personality traits related to neuroticism may interact with the visuo-vestibular system, which is tightly linked with experience of SS (e.g., Bles et al., 1998; Duh et al., 2004). Finally, a more neurotic personality may be predisposed to report their negative symptomatology, as suggested by previous investigations (see Williams and Wiebe, 2000).

#### Types of Environments and Gender Differences

Figure 2C indicates that experiments using realistic (or 360-degree images/videos) scenarios did not often report female susceptibility for VR discomfort and SS. On the other hand, in most of the studies that utilized video-game scenarios or cognitive tasks, the trend was the opposite, with females more often reporting more discomfort than males.

Munafo et al. (2017) performed two experiments; however, they found gender differences in SS only in the second one. They explained the different results by arguing that gender imbalance in SS may depend on the utilized environment, and that such a difference may be hindered if an environment provokes a low level of SS. Furthermore, they suggested that sex differences in SS susceptibility may be differentially related to sex differences in cognitive functions depending on the VR environment (e.g., Voyer et al., 1995; Kimura, 1997; Giammarco et al., 2015), or may have to do with biological differences between males and females (sexual dimorphism). However, the interpretations of the heterogeneous results of Munafo et al. (2017) may only apply to certain types of VR environments (e.g., when postural stability is requested). This eventuality may explain the different female SS prevalence among experiments that employ very different types of scenarios [but see Robert et al. (2016), which did not find gender differences in postural tasks in VR]. VR scenarios that propose games or tasks may require more postural stability due to the relatively high level (compared to the realistic/360-degree scenes that generally do not require any task to be performed, or the tasks are quite simple) of body/hand movements.

Furthermore, gender differences may be more simply due to adaptation. One of the major features of the connected phenomenon of motion sickness is that subjective symptoms of discomfort tend to fade with repeated exposure to a nauseogenic stimulus (Munafo et al., 2017). A prior (more often) reported use of videogames by males compared to females may have reduced male susceptibility to SS and discomfort during the VR experience of a game. This phenomenon has been reported in the literature (Knight and Arns, 2006). Finally, those studies that have employed games are also those generally more emotional/arousing, and this may have also had a role in the female predisposition in developing SS symptomatology.

#### Locomotion, VR-Induced Level of SS, and Gender Differences

The type of locomotion has been often associated with SS, with an environment displaying more locomotion being claimed to enhance SS. SS-inducing properties of virtual locomotion are commonly reported (Bruder et al., 2012; Nilsson et al., 2018). We attempted to understand if locomotion type may have something to do in gender differences of SS. From the two studies of Munafo et al. (2017), it seems that the ability to move through the environment using a joypad may increase the likelihood of female participants to experience higher SS compared to males. At the same time, the study seems to support the claim that an environment promoting a generally high level of SS may negatively affect female participants more than male participants. However, from the studies analyzed in the current literature, it seems that this may not always be the case. For example, the study of Al Zayer et al. (2019) reported no difference between the male and female level of SS, while using an environment inducing a high level of SS (mean SSQ score of 51), and the possibility for the participants to move through the environment with a joypad. However, it is worthy to note that in Munafo et al. (2017) exp 2 (where the difference in SS between males and females was reported), the average level of SS was very high (around 70, an extremely high score if based on previous studies reporting results for the SSQ; see Kennedy et al., 1993). Therefore, it is possible that in an environment inducing an extreme level of induced SS, female participants may be more susceptible. However, this hypothesis is speculative due to little evidence; future studies should aim to investigate the issue further.

### Cognition and Performance Differences

Data regarding gender differences in cognition and performances are heterogeneous, with the majority of the studies not finding any differences in, for example, memory capacity (Cárdenas-Delgado et al., 2017), image quality perception (Juan et al., 2018), cognitive abilities such as information recognition and retrieval as well as cognitive load during the simulation (Roettl and Terlutter, 2018), sense of presence and performance (Khashe et al., 2018), and enjoyment or interest (Rangelova and Marsden, 2018).

However, a handful of studies have found that females perform better than males, for example, a velocity stereovision task (Allen et al., 2016) and localization and memory tasks (Liang et al., 2019). Females were also found to be more sensitive to emotional information in the simulated environment (Mousas et al., 2018), and to report a higher level of sense of embodiment (Scheibler and Rodrigues, 2018), as well as of presence, when 3D images were showed (Narciso et al., 2019). It has been often proposed that sense of presence and performance are often connected (e.g., Lombard and Ditton, 1997; Witmer and Singer, 1998; Grassini et al., 2020). The fact that females perform better may be due to the higher sense of presence (or a presence-related phenomenon such as realism) that female participants sometimes experience (see Bracken, 2005). However, women were not always found to experience a higher sense of presence compared to men (e.g., Gamito et al., 2008); such a phenomenon may be dependent on the content of the VR simulation and/or other uncontrolled experimental variables.

Only one study found an advantage in a learning-related task for males (An et al., 2018). Nevertheless, the context could help to understand the difference in performance in some specific cases. For example, in the study of An et al. (2018), the participants performed a military simulation scenario that, according to general knowledge, may be more interesting for young males compared to young females. This different level of interest may have affected the learning performance.

Even though the papers analyzed in the present literature review generally do not support the existence of gender difference in performance difference during HMD-mediated VR, it is worth noting that these articles are only a subsample of the vast literature on the topic. Cognitive difference between males and females is a widely studied topic, and a large literature has been accumulating over the years (see e.g., Wai et al., 2010). Our attempt was to isolate possible systematic gender differences specifically in the literature of modern HMD-mediated VR. Furthermore, our attempt aimed to stimulate a debate and future lines of research on gender differences in modern technologies that did look not only at SS and ill-related symptoms but also at performance metrics, use possibilities, and adoption of the technology in applied fields.

Please also note that studies focused on gender difference during VR experience have reported gender inequalities in performance metrics. For example, male participants have been often reported to significantly outperform females in the navigation of virtual environments (see e.g., Waller et al., 1998). Furthermore, the widely cited work of Czerwinski et al. (2002) proposed that gender discrepancies in performance during VR simulation might be due to difference in perception of field-of-view, and that field-of-view restriction may impact women negatively more than men—and women take a wider field of view to reach the same performance than men in a navigation task.

### Limitations and Future Studies

The fast development of new technologies complicates the generalization of the present results for past and future types of HMD equipment. For example, even though the present review analyzed studies from 2016, it includes quite a bit of data from experiments that utilized beta versions of HMD products (Oculus DK1 and 2). The results obtained from those obsolete and non-consumer-ready technologies may be different from those that would be obtained from more updated tools.

Furthermore, the relatively specific focus of the present study (i.e., modern HMD technologies), as well as the relatively recent introduction of these technologies to the market, makes the study of this topic challenging. The published scientific literature is limited, and the article sample size analyzed in the present review is not large enough for us to be able to generalize the findings of the present review. Furthermore, we categorized the content of the VR studies, as presented in Table 1, but different investigators may have placed them in different categories (e.g., as emotional/non-emotional VR environments). The level of subjectivity that we had to employ in categorizing the studies is a limitation of the results presented in the review.

The age of the participants of the analyzed studies was generally very low, as most of these empirical studies have used students as experimental subjects. This fact may be a very important limitation for the generalizability of the reported results, as older male and female participants may have a different susceptibility to SS compared to younger ones. Especially with regard to the hormonal theory (related to the female menstrual cycle), older participants may experience different levels of SS depending on their age-related hormonal development.

A further limitation of the present study is that the vast majority of the analyzed studies used environments in which SS is somehow mitigated (e.g., alternative locomotion), and those methods for mitigating SS may interact with gender. However, only a very limited number of papers aimed specifically in inducing a high level of SS, and therefore, it is difficult to draw any conclusion on the issue. Therefore, the implication of the present study might apply mainly to those recently developed VR environments that use methods for SS reduction.

In line with the framework proposed by Saredakis et al. (2020), future studies should aim to utilize quantitative methods to establish and eventually quantify the gender differences in susceptibility to SS. An effort should also be made to differentiate the various aspects of SS, for example, utilizing the SSQ subscales (oculomotor, nausea, disorientation; Kennedy et al., 1993), as a gender difference may be on different SS symptoms and not in the overall report of symptoms degree.

Finally, the types of studies analyzed in the present review were extremely heterogeneous: these presented a number of research design with different problems (e.g., limited number of participants in the experiment, or gender unbalance of the participants). These differences need to be taken into consideration when evaluating the robustness of the results. For example, the study of Narciso et al. (2019) used a “quasi-experimental design” and excluded 23% of their sample as being “outliers” (without providing an adequate explanations of the exclusion criteria). However, the authors of the present review have aimed in presenting possible strength and limitation of the analyzed studies in an objective way to the reader (presenting, for example, the number of participants of each of the study), without establishing criteria (possibly including a bias) for evaluating the quality of the included studies. However, please note that the current review is limited by the fact that the reviewed studies were not assessed for their quality.

## Conclusion

The results of the present review showed that it is difficult to establish an overall consensus in literature for a gender difference in SS susceptibility; most of the analyzed studies did not find any association between gender and SS. Furthermore, it seems that most of the studies that reported gender differences utilized particular types of simulation environments (e.g., video games) or had certain types of contents (emotional/arousing contents or tasks). It is possible that if a gender difference exists in experiencing different types of HMD-mediated VR environment: (1) it may have a smaller effect size than previously thought; (2) it may only affect certain aspects of SS (e.g., only nausea-related or oculomotor-related symptoms); or (3) the effect may be not generalized to the entire female population (susceptibility that depends on other factors but not biological sex *per se*). A complex combination of different individual factors and uncontrolled experimental variables may best explain the discrepancies of the results found in the literature.

Other analyzed sex differences between male and female participants—for example, cognitive ability in VR settings and task performance—generally did not differ between genders. However, some studies have reported better task performance for female compared to male participants. We propose that such difference may be due to a higher sense of presence sometimes reported by female participants in HMD-mediated VR.

The heterogeneous results reported in the present systematic review diminish the hypothesis that claims the existence of generalized “sexist” aspects of human experience in HMD technology. However, future scientific efforts should attempt to quantify the effect size of a possibly higher susceptibility of female participants to SS. The results of the present investigation should also encourage female users to try HMD technology, without fearing that this action may have a high probability of causing them discomfort. A higher level of adoption of the technology in females may also push developers and industries to focus on producing equipment that is more suitable for female use (as in the case of an IPD adjustment mechanism).

## Data Availability Statement

The original contributions presented in the study are included in the article/supplementary material, further inquiries can be directed to the corresponding author/s.

## Author Contributions

SG performed the review, elaborated the selection process for the included articles, and wrote the draft of the present manuscript. KL commented and gave her inputs and suggestions on the draft of the manuscript and helped develop and conceptualize the study idea. Both authors approved the final version of the manuscript prior to submission.

## Conflict of Interest

The authors declare that the research was conducted in the absence of any commercial or financial relationships that could be construed as a potential conflict of interest.
